# Time since SARS-CoV-2 infection and humoral immune response following BNT162b2 mRNA vaccination

**DOI:** 10.1016/j.ebiom.2021.103589

**Published:** 2021-09-24

**Authors:** Brent Appelman, Karlijn van der Straten, A.H. Ayesha Lavell, Michiel Schinkel, Marleen A. Slim, Meliawati Poniman, Judith A. Burger, Melissa Oomen, Khadija Tejjani, Alexander P.J. Vlaar, W. Joost Wiersinga, Yvo M. Smulders, Lonneke A. van Vught, Rogier W. Sanders, Marit J. van Gils, Marije K. Bomers, Jonne J. Sikkens, Amsterdam UMC COVID-19 S3/HCW study group, Brent Appelman, Diederik van de Beek, Marije K Bomers, Justin de Brabander, Matthijs C Brouwer, David TP Buis, Nora Chekrouni, Marit J van Gils, Menno D de Jong, AH Ayesha Lavell, Niels van Mourik, Sabine E Olie, Edgar JG Peters, Tom DY Reijnders, Michiel Schinkel, Alex R Schuurman, Jonne J Sikkens, Marleen A Slim, Karlijn van der Straten, Yvo M Smulders, Alexander PJ Vlaar, Lonneke A van Vught, W Joost Wiersinga

**Affiliations:** a Center for Experimental and Molecular Medicine, Amsterdam Infection & Immunity, AMC; b Neurologie, Neuroscience, AMC; c Interne Geneeskunde, Amsterdam Infection & Immunity, VUmc; d Medical Microbiology & Infection , Amsterdam Infection & Immunity, AMC; e Intensive care medicine, Amsterdam Infection & Immunity, AMC; f Intensive care medicine & Center for Experimental and Molecular Medicine, Amsterdam Infection & Immunity, AMC; a Center for Experimental and Molecular Medicine, Amsterdam UMC, Amsterdam Institute for Infection and Immunity, University of Amsterdam, Meibergdreef 9, Amsterdam 1105 AZ, the Netherlands; b Department of Medical Microbiology, Amsterdam UMC, Amsterdam Institute for Infection and Immunity, University of Amsterdam, Meibergdreef 9, Amsterdam 1105 AZ, the Netherlands; c Department of Internal Medicine, Amsterdam UMC, Amsterdam Institute for Infection and Immunity, Vrije Universiteit Amsterdam, De Boelelaan 1117, Amsterdam 1081 HV, the Netherlands; d Department of Intensive Care Medicine, Amsterdam UMC, Amsterdam Institute for Infection and Immunity, University of Amsterdam, Meibergdreef 9, Amsterdam 1105 AZ, the Netherlands; e Department of Internal Medicine, Amsterdam UMC, Amsterdam Institute for Infection and Immunity, University of Amsterdam, Meibergdreef 9, Amsterdam 1105 AZ, the Netherlands; f Department of Microbiology and Immunology, Weill Medical College of Cornell University, New York, USA

**Keywords:** Vaccine, BNT162b2, SARS-CoV-2, COVID-19, Humoral immune response, Neutralisation

## Abstract

**Background:**

To optimise the use of available SARS-CoV-2 vaccines, some advocate delaying second vaccination for individuals infected within six months. We studied whether post-vaccination immune response is equally potent in individuals infected over six months prior to vaccination.

**Methods:**

We tested serum IgG binding to SARS-CoV-2 spike protein and neutralising capacity in 110 healthcare workers, before and after both BNT162b2 messenger RNA (mRNA) vaccinations. We compared outcomes between participants with more recent infection (*n* = 18, median two months, IQR 2-3), with infection-vaccination interval over six months (*n* = 19, median nine months, IQR 9-10), and to those not previously infected (*n* = 73).

**Findings:**

Both recently and earlier infected participants showed comparable humoral immune responses after a single mRNA vaccination, while exceeding those of previously uninfected persons after two vaccinations with 2.5 fold (*p* = 0.003) and 3.4 fold (*p* < 0.001) for binding antibody levels, and 6.4 and 7.2 fold for neutralisation titres, respectively (both *p* < 0.001). The second vaccine dose yielded no further substantial improvement of the humoral response in the previously infected participants (0.97 fold, *p* = 0.92), while it was associated with a 4 fold increase in antibody binding levels and 18 fold increase in neutralisation titres in previously uninfected participants (both *p* < 0.001). Adjustment for potential confounding of sex and age did not affect these findings.

**Interpretation:**

Delaying the second vaccination in individuals infected up to ten months prior may constitute a more efficient use of limited vaccine supplies.

**Funding:**

Netherlands Organization for Health Research and Development ZonMw; Corona Research Fund Amsterdam UMC; Bill & Melinda Gates Foundation.

**Research in context**

**Evidence before this study**

Recent studies show that a single mRNA vaccination in individuals with recent COVID-19 (up to six months prior) provides a potent immune response, equivalent to, or exceeding, the antibody response after two vaccinations in individuals without previous SARS-CoV-2- infection. Little is known about immune responses after a single vaccine dose in individuals that suffered from COVID-19 over six months prior to vaccination.

**Added value of this study**

We show that one dose of the BNT162b2 mRNA vaccine induces a humoral immune response in individuals previously infected with SARS-CoV-2 that exceeds antibody responses in uninfected individuals after two vaccine doses, even if the infection occurred more than six months prior. The humoral immune response after each dose in individuals infected over six months prior was shown to be at least similar to those recently infected; the second vaccination elicited no substantial improvement of humoral response for previously infected in either group. Our study is the first to compare data of individuals with recent infection (within six months) to those infected over six months ago - and suggests a single mRNA vaccine in individuals infected up to ten months prior to vaccination is sufficient to elicit a potent humoral immune response.

**Implications of all the available evidence**

To maximise the number of individuals protected against SARS-CoV-2 by vaccination, delayed administration of the second dose for individuals with previous infection up to six months is accepted policy in parts of the world. Available evidence suggests this strategy could include individuals that suffered COVID-19 up to ten months prior to vaccination, and possibly longer. This could enable earlier vaccination of uninfected individuals.

Alt-text: Unlabelled box

## 1 Introduction

Since December 2020 over one billion vaccines have been administered worldwide as the main strategy to combat this Severe Acute Respiratory Syndrome Coronavirus 2 (SARS-CoV-2) pandemic by inducing artificial herd immunity [1]. The rate-limiting factor for many vaccine strategies is the limited availability of vaccines. Studies on antibody response following vaccination are emerging, and demonstrate that for those with previous SARS-CoV-2 infection one dose of messenger RNA (mRNA) vaccine induces antibody levels similar to, or even exceeding the antibody response for those without previous SARS-CoV-2 infection after two doses [2], [3], [4], [5], [6], [7], [8]. Data on antibody response in patients with a long interval between infection and vaccination (i.e. more than six months) are still sparse. We compared the SARS-CoV-2 Spike protein specific IgG antibody levels and neutralising antibody titres of sera before and after the first and second dose of BNT162b2 (Pfizer-BioNTech) mRNA vaccine between (1) participants infected within six months prior to vaccination, (2) previously infected participants infected earlier (over six months prior to vaccination) and (3) previously uninfected participants.

## 2 Methods

### 2.1 Study design

In March 2020 we initiated a prospective serologic surveillance cohort study among hospital healthcare workers in two tertiary medical centers in the Netherlands (S3 cohort; NL 73478.029.20, Netherlands Trial Register NL8645). In short, follow-up visits were scheduled regularly (March, April, May, June, October 2020, January 2021) and included serological testing, surveys regarding results of nucleic acid amplification testing (NAAT), and presence of COVID-19 related symptoms since the previous visit. For comprehensive details about inclusion and follow-up of this cohort we refer to the original article of the S3 study [9].

Between January 6th and 13th 2021, a selection of cohort participants received their first dose of BNT162b2 mRNA vaccine. This selection was based on potential, work-related, high exposure to SARS-CoV-2 as part of the national vaccination strategy in the Netherlands. A second dose was administered 21 days after the first; sera were obtained within 24 h of the first vaccination, 21 days after the first vaccination, and 28 days after the second dose.

Participants were divided in three groups with regard to previous infection status: (1) participants infected within six months prior to vaccination, named recently infected, (2) participants infected earlier (over six months prior to vaccination), named earlier infected, and (3) previously uninfected participants.

The infection date was determined by the date of a positive SARS-CoV-2 NAAT result. For subjects without an available positive NAAT result, the timing of infection was based on the history of clinical symptoms in combination with seroconversion measured at previous timepoints of the study. For participants with asymptomatic seroconversion, we used the midpoint between the last seronegative sample and the first seropositive sample. Participants without a positive NAAT result or seroconversion during follow-up since onset of the cohort in March 2020, were considered uninfected.

### 2.2 Ethics

The study was approved by the Medical Research Ethics Committee of both hospitals and accepted by the competent authority, the Central Committee on Research on Human Subjects (NL73478.029.20). Written informed consent was obtained from each participant.

### 2.3 Serological response

In the months prior to vaccination, seroconversion was defined as a serological response using a Wantai SARS-CoV-2 total-Ig enzyme-linked immunosorbent assay (Wantai ELISA) [10]. To quantify the serum IgG response to SARS-CoV-2 spike protein following vaccination, we used a custom Luminex assay. To identify recent SARS-CoV-2 infections, serum obtained within 24 h of first vaccination was tested for a serological response by using the Luminex assay. In case of intermediate IgG SARS-CoV-2 spike protein binding in previously uninfected participants, a Wantai ELISA was performed to confirm this recent infection.

The custom Luminex assay was described previously [11]. In short, prefusion stabilized trimeric SARS-CoV-2 spike protein was covalently coupled to Luminex Magplex beads with a ratio of 75 µg protein to 12.5 million beads. The protein design of SARS-CoV-2 spike protein is described previously [11]. Optimisation studies showed an optimal dilution of sera of 1:100,000 for measuring the infection and vaccination response. After an overnight incubation, plates were washed with TBS containing 0.05% Tween-20 (TBST) and resuspended in 50 µl of Goat-anti-human IgG-PE (RRID AB_2795648, validated by Southern Biotech). Read-out was performed on a Magpix (Luminex). Resulting mean fluorescence intensity (MFI) values are the median of approximately 50 beads per well and were corrected by subtraction of MFI values from buffer and beads only wells.

To investigate the neutralising capacity of sera of those previously infected and a random sample of 50 previously uninfected, we used the previously described pseudovirus neutralisation assay [11]. In short, serial dilutions of heat-inactivated sera were mixed 1:1 with SARS-CoV-2 pseudovirus and incubated for 1h at 37 °C before adding this mixture to HEK293T cells expressing angiotensin converting enzyme 2 (ACE2) receptor of SARS-CoV-2. After 48 h of incubation at 37 °C, cells were lysed and luciferase activity was measured by using Nano-Glo Luciferase Assay System (Promega). Relative luminescence units were normalized to the units from cells infected with pseudovirus in absence of serum. Neutralisation levels were the serum dilution at which infectivity was inhibited 50% (ID_50_) using a non-lineair regression curve fit (GraphPad Prism software version 8.3). Neutralisation ID_50_ values < 100 were considered negative.

### 2.4 Statistical analysis

Binding antibody levels and neutralisation titres were reported as medians with interquartile ranges (IQR). Antibody levels were compared between groups by using a Mann-Whitney-U test (MW). For categorical outcomes a chi-square test was used (χ2). In order to adjust for participants’ sex and age as potential confounders, we log transformed all outcomes and then performed univariable and multivariable linear regression analysis. Results were considered statistically significant at *p* < 0.05. We used R Core Team (2020). R: A language and environment for statistical computing. R Foundation for Statistical Computing, Vienna, Austria.

### 2.5 Role of the funding source

The funders of the study had no role in the study design, data collection, data analysis, writing of the report, or in the decision to submit for publication.

## 3 Results

We included 110 participants who received their first vaccination with BNT162b2 (Pfizer-BioNTech) mRNA vaccine in January 2021, of whom 73 individuals were previously uninfected and 37 had a documented infection with SARS-CoV-2 in the past year. 8 out of 37 participants remained asymptomatic during infection. Median age of participants was 42 years (IQR 32–54) and 69% were female (Table 1). The median interval between SARS-CoV-2 infection and vaccination in the recently infected group was two months (IQR 2-3, *n* = 18), and in the earlier infected group nine months (IQR 9-10, *n* = 19) (Table 1). Binding antibody findings of three participants were excluded from analysis due to technical issues. Neutralising antibody levels of one participant after the first vaccination were excluded because of a suspected sample switch as this sample showed a discrepant high neutralisation capacity compared to normal binding levels at the same time point and lower neutralisation titres following second vaccination.

**Table 1:** Study participants characteristics.

	**Previously infected** **(*n* = 37)**		**Uninfected** **(*n* = 73 )**	**Total (*n* = 110)**
	Recent infection (<6 months) (*n* = 18)	Earlier infection (>6 months) (*n* = 19)		
**Age,** y (median (IQR))	40.0 (32.0-52.0)	32.0 (27.0-42.0)	44.0 (33.0-54.0)	42.0 (32.0-54.0)
**Sex,** Female (%)	12 (67)	18 (95)	46 (63)	76 (69)
**Previous infection documented by:**				
Both NAAT & serology positive (%)	15 (83)	12 (63)		27 (25)
Only seroconversion (%)	3 (17)	7 (37)		10 (9)
**Time between vaccine and infection,** Months (median (IQR))	2.0 (2.0-3.0)	9.0 (9.0-10.0)	-	5.5 (2.0-9.0)

NAAT: nucleic acid amplification testing. IQR: interquartile range.

### 3.1 Humoral immune response in participants with and without previous infection

**Fig. 1:**
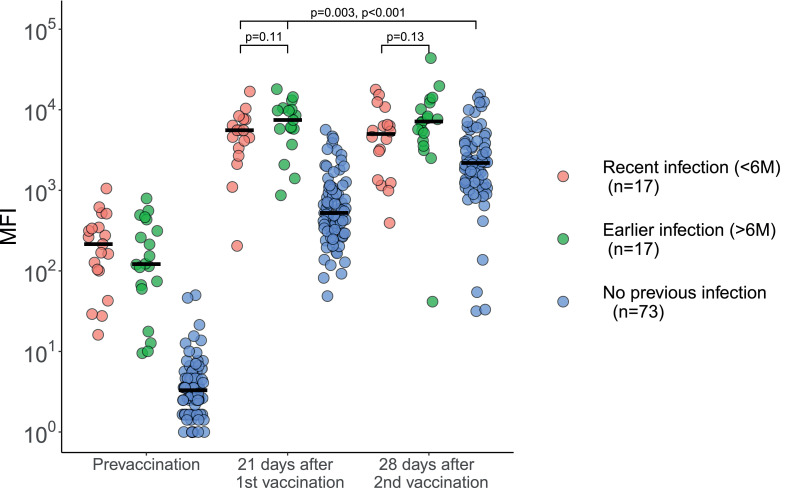

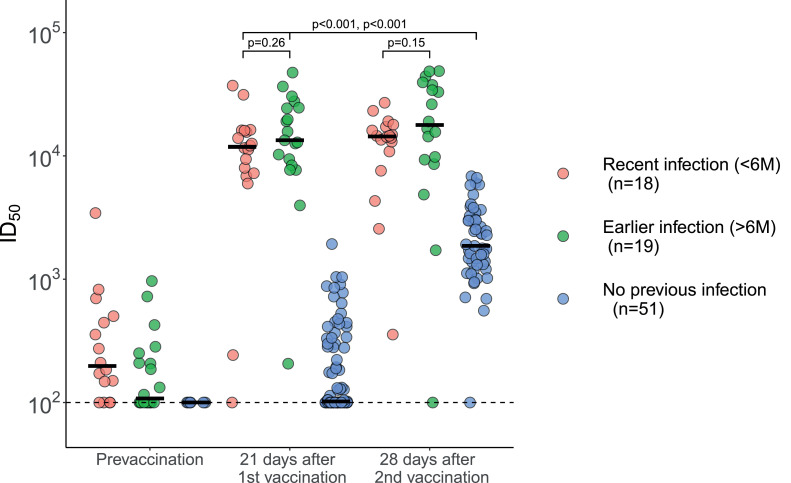
Antibody levels and neutralisation in convalescent COVID-19 patients and uninfected participants prior to and after first and second vaccine dose. The overarching line represents the comparison of both the previous infected groups after the first vaccination with the non-infected group after the second vaccination. The number of patients (n) in the legend is for the prior infectious group on “21 days after vaccination” and for the non-infectious group on “28 days after second vaccination”. a: Serum IgG binding levels to SARS-CoV-2 spike protein. The number of participants with a recent infection, earlier infection and no previous infection pre-vaccination were: *n* = 19, *n* = 20 and *n* = 84, respectively, at 21 days after the first vaccination: *n* = 17, *n* = 17, *n* = 82, respectively, and at 28 days after the second vaccination: *n* = 18, *n* = 19 and *n* = *n* = 73, respectively. Group medians were compared using the Mann-Whitney-U test. There was no significant difference at 21 days after the first vaccination and at 28 days after the second vaccination between the recent infection and the earlier infection groups, *p* = 0•11 and *p* = 0•13, respectively. The results of the recently infected and earlier infected groups at 21 days after the first vaccination were significantly different from the non-infected group at 28 days after the second vaccination, *p* = 0•003 and *p* < 0•001 respectively. MFI: Mean Fluorescence Intensity. b: Serum neutralisation of SARS-CoV-2 pseudovirus, with a lower limit of detection of 100 ID_50_. The number of participants with a recent infection, earlier infection and no previous infection pre-vaccination were: *n* = 16, *n* = 20 and *n* = 6, respectively, at 21 days after the first vaccination: *n* = 18, *n* = 19, *n* = 82, respectively, and at 28 days after the second vaccination: *n* = 18, *n* = 18 and *n* = 51, respectively. Group medians were compared using the Mann-Whitney-U test. There was no significant difference at 21 days after the first vaccination and at 28 days after the second vaccination between the recently infected and earlier infected groups, *p* = 0•26 and *p* = 0•15, respectively. The recently infected and the earlier infected groups at 21 days after the first vaccination were significantly different from the non-infected group at 28 days after the second vaccination, *p* < 0•001 and *p* < 0•001 respectively. ID_50_: 50% Inhibitory Dilution.

Most participants with prior documented SARS-CoV-2 infection still had detectable anti-spike protein antibodies pre-vaccination (median 162 MFI, IQR 70-341, Fig. 1a). After one vaccine dose, binding antibody levels increased 36 and 664 fold for the previously infected and uninfected individuals, respectively. Binding antibody levels in previously infected individuals after one dose significantly exceeded those observed in the fully vaccinated individuals without a history of SARS-CoV-2 infection (median 5846 MFI, IQR 3806-9394, and 2188 MFI, IQR 1200–3848, respectively, *p* < 0.001 (MW)). Similarly, individuals with a previous infection of SARS-CoV-2 had higher neutralising antibody titres after one vaccine dose, compared to fully vaccinated uninfected individuals (median 12,615 ID_50_, IQR 8003–19,111, and 1863 ID_50_, IQR 1314–3031, respectively, *p* < 0.001 (MW)) (Fig. 1b). Only a few uninfected individuals showed substantial neutralising titres following a single vaccination (median 102 ID_50_, IQR 100–315). None of the participants had signs of an SARS-CoV-2 infection after the first vaccination.

After the second vaccine dose, binding antibody levels and neutralisation titres increased 4.1 and 18.2 fold for the previously uninfected individuals, respectively; whereas antibody binding levels and neutralisation titres in previously infected individuals did not change substantially: 0.92 and 1.17 fold change, respectively.

### 3.2 Adjustment for confounding

Univariable regression analysis mirrored above outcomes: binding antibody levels values were significantly higher in both the recently infected group (log MFI: 8.33) and earlier infected group (log MFI: 8.72) 21 days after the first vaccination, as compared to the uninfected group 28 days after the second vaccination (log MFI: 7.59, difference 0.74, 95% CI: 0.15–1.34 and 1.13 ,95% CI: 0.53–1.72, respectively). Neutralisation titres were significantly higher in both the recently infected group (log ID_50:_ 8.96) and earlier infected group (log ID_50:_ 9.41) 21 days after the first vaccination, as compared to the uninfected group 28 days after the second vaccination (log ID_50:_ 7.57, difference 1.39 (95% CI: 0.81–1.95) and 1.84 (95% CI: 1.28–2.39), respectively). Adding sex and age to the model as potential confounders, did not importantly alter these results (Table 2).

**Table 2:** Univariable and multivariable linear regression analysis. Serum binding antibody levels in log transformed MFI values 21 days after the first vaccination in recently (*n* = 17) and earlier infected participants (*n* = 17), were compared to log transformed MFI values 28 days after the second vaccination in uninfected participants (*n* = 73). Neutralizing capacity in log transformed ID_50_ values in recently (*n* = 18) and earlier infected participants (*n* = 19) were compared to log transformed ID_50_ values 28 days after the second vaccination in uninfected participants (*n* = 51). Multivariable model includes participant sex and age as potential confounders.

	**log MFI**	**Difference with uninfected (95% CI)**	**log ID50**	**Difference with uninfected (95% CI)**
**Univariable**				
Uninfected	7.59	-	7.57	-
Recently infected	8.33	0.74 (0.15–1.34)	8.96	1.39 (0.82–1.95)
Earlier infected	8.72	1.13 (0.53-1.72)	9.41	1.84 (1.28–2.39)
**Multivariable**				
Uninfected	7.63	-	7.24	-
Recently infected	8.31	0.68 (0.09–1.26)	8.60	1.36 (0.80–1.92)
Earlier infected	8.48	0.85 (0.22–1.47)	8.91	1.67 (1.09–2.25)

CI: Confidence interval, MFI: Mean Fluorescence Intensity, ID_50_: 50% Inhibitory Dilution.

### 3.3 Time since infection and humoral immune response

Pre-vaccination antibody binding levels were similar between recently and earlier infected participants (median 215 MFI, IQR 65–343, and 121 MFI, IQR 62–404, *p* = 0.33 (MW)). After the first vaccination dose, no difference was observed between these groups for both binding antibody levels (5,558 MFI, IQR 3353-7,584, and 7453 MFI, IQR 5788-10,062, *p* = 0.11 (MW)), and neutralisation titres (11,844 ID_50_, IQR 7428–15,924 and 13,384 ID_50_, IQR 8907–24,475, *p* = 0.26 (MW)). After the second vaccination, antibody binding levels were similar between recently and earlier infected participants (median 4989 MFI, IQR 1770–6479, and median 7131 MFI, IQR 4628–11,213, *p* = 0.13 (MW)). Corresponding neutralisation titres were: median 14,391 ID_50_, IQR 11,409–16,931, and 17,832 ID_50_, IQR 9447–36,747, *p* = 0.15 (MW).

Binding antibody levels and neutralising titres are plotted against time since infection in Supplementary Fig. S1a + b.

### 3.4 Side effects

Overall, 100 (91%) participants reported any side effect after the first dose, and 84 (76%) after the second dose. After the first vaccination dose, previously infected individuals experienced more local skin reactions (19.4 vs 2.9%, *p* = 0.01 (χ2)) and muscle soreness (61.1 vs 24.3%, *p* < 0.001 (χ2)) compared to uninfected individuals. Also, more individuals without prior infection reported no complaints at all (0 vs 14.3%, *p* = 0.04 (χ2)). We found no significant difference in the incidence of side effects after the second dose (Supplementary Table S1).

## 4 Discussion

In this study, we demonstrate that one dose of the BNT162b2 mRNA vaccine boosts the humoral immune response in individuals previously infected with SARS-CoV-2 to a level that exceeds antibody responses in uninfected individuals after two vaccine doses, even if the infection occurred over six months prior.

The humoral immune response after each dose in individuals infected over six months ago was at least similar to those recently infected. In addition, no substantial rise in serum binding antibody levels or neutralising capacity was observed following second vaccination in either group of previously infected individuals. Our study is the first to compare SARS-CoV-2 antibody binding and neutralisation responses in individuals with recent infection (within six months) to those infected more than six months prior to vaccination. Also it is the first to include all relevant time points (before vaccination, 21 days after first vaccination, 28 days after second vaccination), in a well documented cohort followed since the onset of the pandemic.

Our findings are in line with previous studies showing that recently infected participants (mean time since infection: 111 days) had higher neutralisation titres after one vaccine dose in comparison to previously uninfected participants after the second dose, with a trend towards increasing neutralisation titres over time since infection [7]. Another study comprising only individuals with earlier infection (median time since infection: eight-nine months) showed both higher binding and neutralising antibody responses after a single dose of mRNA vaccine compared to individuals not previously infected [6]. These studies together with the current study strongly suggest that a single vaccine dose in previously infected individuals with an infection-vaccine interval longer than six months induces an immune response at least similar to recently infected individuals. This conclusion is in line with the hypothesis that infection is analogous to a first vaccine dose, making the first real vaccine dose act as a ‘boost’ for individuals with a history of SARS-CoV-2 infection [4]. This response in previously exposed individuals is most likely explained by a recall of SARS-CoV-2 specific memory B cells elicited during their first exposure to the virus [12]. Our results demonstrate this effect is durable over time up to at least ten months after infection.

Furthermore, we found no substantial change in immune response following the second vaccination for previously infected participants. Prior research found serum neutralising potency against SARS-CoV-2 pseudovirus actually decreased following the second dose in previously infected individuals, reducing the likelihood of additional benefit of the second dose in these individuals.^2^

Considering limited vaccine supply in the midst of this global pandemic, several countries (including the Netherlands) currently recommend administering a single dose to individuals infected in the previous six months, whilst the regular scheme of two doses is advised when infection was over six months ago [13].^12^ Our results suggest this may be extended to at least ten months past infection, which could make vaccines for previously uninfected individuals more readily available.

Our study has some important potential limitations. First and foremost the sample size of previously infected participants is relatively low. Second, our healthcare worker cohort consists of relatively healthy, young individuals with a mild or asymptomatic history of SARS-CoV-2 infection; results may not be generalisable to e.g. immunocompromised individuals or those with severe previous COVID-19 disease. Lastly, we did not evaluate cellular immune responses which are likely to contribute to vaccine efficacy as well [14]. However, neutralising antibody levels are shown to be predictive of immunity to COVID-19 [15].

In conclusion, one dose of the BNT162b2 mRNA vaccine induces humoral immune responses in individuals previously infected with SARS-Cov-2 exceeding those of uninfected individuals after two doses, whether infected occurred recently or over six months prior to vaccination. Delayed administration of the second vaccination dose for individuals with previous infection up to ten months, and likely longer, may constitute a more efficient vaccination strategy.

## 5 Study group

**Table:** 

First Name	Surname	Degree	Department	Research institue	Location
Brent	Appelman	Dhr, MD	Center for Experimental and Molecular Medicine	Amsterdam Infection & Immunity	AMC
Diederik	van de Beek	Dhr, MD PhD	Neurology	Amsterdam Neuroscience	AMC
Marije K	Bomers	Mw, MD PhD	Internal medicine	Amsterdam Infection & Immunity	VUmc
Justin	de Brabander	Dhr, MD	Center for Experimental and Molecular Medicine	Amsterdam Infection & Immunity	AMC
Matthijs C	Brouwer	Dhr, MD PhD	Neurology	Amsterdam Neuroscience	AMC
David TP	Buis	Dhr, MD	Internal medicine	Amsterdam Infection & Immunity	VUmc
Nora	Chekrouni	Mw, MD	Neurology	Amsterdam Neuroscience	AMC
Marit J	van Gils	Mw, PhD	Medical Microbiology & Infection prevention	Amsterdam Infection & Immunity	AMC
Menno D	de Jong	Dhr, MD PhD	Medical Microbiology & Infection prevention	Amsterdam Infection & Immunity	AMC
AH Ayesha	Lavell	Mw, MD	Internal medicine	Amsterdam Infection & Immunity	VUmc
Niels	van Mourik	Dhr, MD	intensive care medicine	Amsterdam Infection & Immunity	AMC
Sabine E	Olie	Mw, MD	Neurology	Amsterdam Neuroscience	AMC
Edgar JG	Peters	Dhr, MD PhD	Internal medicine	Amsterdam Infection & Immunity	VUmc
Tom DY	Reijnders	Dhr, MD	Center for Experimental and Molecular Medicine	Amsterdam Infection & Immunity	AMC
Michiel	Schinkel	Dhr, MD	Center for Experimental and Molecular Medicine	Amsterdam Infection & Immunity	AMC
Alex R	Schuurman	Dhr, MD	Center for Experimental and Molecular Medicine	Amsterdam Infection & Immunity	AMC
Jonne J	Sikkens	Dhr, MD PhD	Internal medicine	Amsterdam Infection & Immunity	VUmc
Marleen A	Slim	Mw, MD	Intensive care medicine	Amsterdam Infection & Immunity	AMC
Karlijn	van der Straten	Mw, MD	Medical Microbiology & Infection prevention	Amsterdam Infection & Immunity	AMC
Yvo M	Smulders	Dhr, MD PhD	Internal medicine	Amsterdam Infection & Immunity	VUmc
Alexander PJ	Vlaar	Dhr, MD PhD	Intensive care medicine	Amsterdam Infection & Immunity	AMC
Lonneke A	van Vught	Mw, MD PhD	Intensive care medicine & Center for Experimental and Molecular Medicine	Amsterdam Infection & Immunity	AMC
W Joost	Wiersinga	Dhr, MD PhD	Center for Experimental and Molecular Medicine	Amsterdam Infection & Immunity	AMC

## Contributor

All contributing authors have read and approved the final version of the manuscript.

BA, KvdS and AHAL (equal): Conceptualisation, data curation and verification, formal analysis, investigation, methodology, project administration, visualisation, writing - original draft and writing - review & editing.

MSch and Mslim: Investigation, project administration, writing - review & editing

MP, JB, MO, KT: data curation, formal analysis, investigation, project administration.

AV, WW, RS and YS: Conceptualisation, investigation, project administration, resources, writing - review & editing.

LV: Conceptualisation, data curation investigation, project administration, resources, writing - review & editing.

MG: Conceptualisation, formal analysis, investigation, project administration, resources, supervision, writing - review & editing.

MB and JS: Conceptualisation, data verification, formal analysis, funding acquisition, investigation, methodology, project administration, resources, supervision, validation, writing - original draft, writing - review & editing.

## Data sharing statement

The original study protocol and data dictionary will be made available to researchers upon request. Researchers willing to access the de-identified participant dataset should send a request to j.sikkens@amsterdamumc.nl. Requests for data will be evaluated and access will depend on the informed consent and permission of legal research support of Amsterdam UMC.

## Declaration of Competing Interest

M. Bomers and J. Sikkens report grants from Netherlands Organization for Health Research and Development ZonMw, grants from Amsterdam UMC Corona Research Funds, during the conduct of the study. All other authors declare no conflict of interests.

## Appendix Supplementary materials

Image, application 1

Image, application 2
