# Supplementary material for: Time since SARS-CoV-2 infection and humoral immune response following BNT162b2 mRNA vaccination
Source: eBioMedicine. 2021 Sep 24;72:103589. doi: 10.1016/j.ebiom.2021.103589 (PMC8461365; doi:10.1016/j.ebiom.2021.103589)
Supplement: Supplementary file 1 [file mmc1.docx]

**Caption supplementary material**

**Supplementary table S1.** Side-effects after first and second vaccination for participants with and without previous infection.


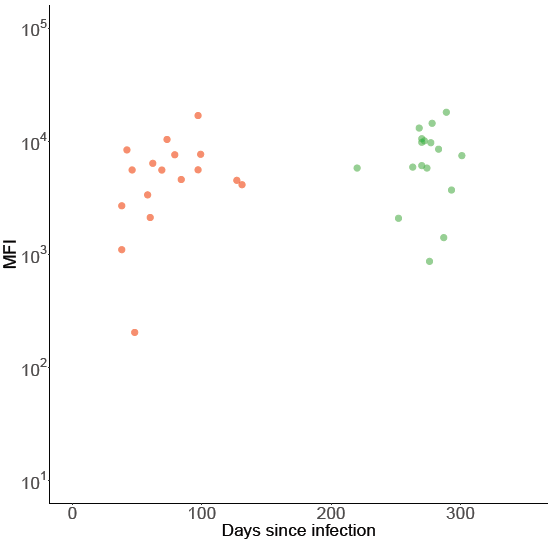


**Supplementary figure S1a**. Serum IgG binding levels to SARS-CoV-2 spike protein after first vaccine dose in convalescent participants by days since infection. MFI: Mean Fluorescence Intensity.


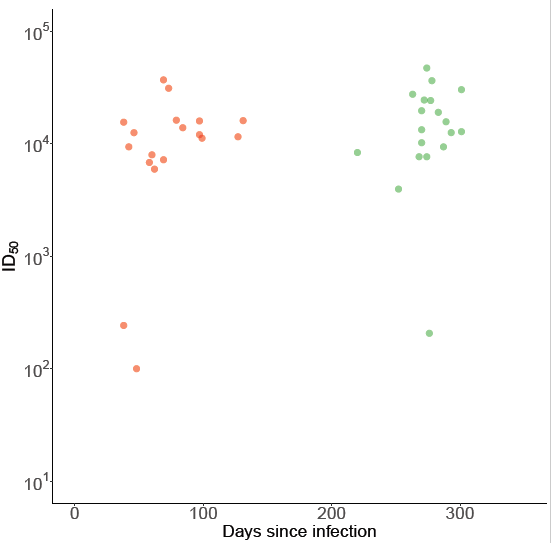


**Supplementary figure S1b**. Serum neutralisation of SARS-CoV-2 pseudovirus after first vaccine dose in convalescent participants by days since infection. ID_50_: 50% Inhibitory Dilution.
