# Supplementary material for: Time since SARS-CoV-2 infection and humoral immune response following BNT162b2 mRNA vaccination
Source: eBioMedicine. 2021 Sep 24;72:103589. doi: 10.1016/j.ebiom.2021.103589 (PMC8461365; doi:10.1016/j.ebiom.2021.103589)
Supplement: Supplementary file 2 [file mmc2.docx]

|  | **Prior infection (n=37)** | **No prior infection (n=73)** |  | **Prior infection (n=37)** | **No prior infection (n=73)** |  |
| --- | --- | --- | --- | --- | --- | --- |
|  | **1st vaccination** | **1st vaccination** | **p-value** | **2nd vaccination** | **2nd vaccination** | **p-value** |
| **Pain (%)** | 30 (83·3) | 47 (67·1) | 0·12 | 13 (35·1) | 29 (39·7) | 0·79 |
| **Skin reaction (%)** | 7 (19·4) | 2 (2·9) | 0·01* | 2 (5·4) | 5 (6·8) | 1 |
| **Skin leasion in cm (mean (SD))** | 1·86 (1·21) | 2·00 (1·41) | 0·89 | 1·50 (0·71) | 2·40 (0·89) | 0·27 |
| **Muscle soreness (%)** | 22 (61·1) | 17 (24·3) | <0·001* | 11 (29·7) | 27 (37·0) | 0·59 |
| **Fatigue (%)** | 9 (25·0) | 12 (17·1) | 0·48 | 5 (13·5) | 21 (28·8) | 0·12 |
| **Fever (%)** | 2 (5·6) | 0 (0·0) | 0·22 | 4 (10·8) | 11 (15·1) | 0·75 |
| **Headache (%)** | 12 (33·3) | 13 (18·6) | 0·15 | 10 (27·0) | 21 (28·8) | 1 |
| **No complaint (%)** | 0 (0·0) | 10 (14·3) | 0·04* | 7 (18·9) | 19 (26·0) | 0·55 |
| **Doctor visit (%)** | 5·6 | 0 | 0·29 | 4·5 | 1·9 | 1 |
| **Self-reported nuisance in case of symptoms (1-10) (median [IQR])** | 1·50 [1·00, 3·00] | 2·00 [1·00, 5·00] | 0·09 | 6·00 [5·75, 7·25] | 4·00 [2·00, 6·00] | 0·08 |
